# Analysis of SARS-CoV-2 Cases, COVID-19 Outcomes and Vaccinations, during the Different SARS-CoV-2 Variants in Greece

**DOI:** 10.3390/vaccines11010126

**Published:** 2023-01-04

**Authors:** Foteini Malli, Ioannis C. Lampropoulos, Garifallia Perlepe, Dimitrios Papagiannis, Konstantinos I. Gourgoulianis

**Affiliations:** 1 Respiratory Disorders Laboratory, Faculty of Nursing, University of Thessaly, 382 21 Larissa, Greece; 2 Respiratory Medicine Department, Faculty of Medicine, University of Thessaly, 382 21 Larissa, Greece; 3 Public Health & Vaccines Laboratory, Faculty of Nursing, School of Health Sciences, University of Thessaly, 382 21 Larissa, Greece

**Keywords:** COVID-19, SARS-CoV-2, variant, Omicron, Delta, mortality, morbidity

## Abstract

Since the emergence of the SARS-CoV-2 Omicron variant, many issues have arisen. We report SARS-CoV-2 vaccinations, SARS-CoV-2 cases and COVID-19 outcomes in Greece during weeks 2–26 of 2021 (Alpha variant period), weeks 27–51 of 2021 (Delta variant period) and week 51 of 2021 to week 27 of 2022 (Omicron variant period). The average weekly cases were higher during the Omicron period vs. the Delta (25,354.17 cases/week) and Alpha periods (11,238.48 cases/week). The average weekly vaccinations were lower in the Omicron period (26,283.69/week) than in the Alpha and Delta period. Joinpoint regression analysis identified that the trend of SARS-CoV-2 cases increased by 88.5% during the rise of the Omicron wave in Greece. The trend of the intensive care unit (ICU) admissions related to COVID-19 decreased by 5.0% immediately after the rise of Omicron while the trend of COVID-19-related deaths decreased by 8.1% from the 5th week of the Omicron wave until the end of the study. For vaccinations, an increasing trend of 8.3% was observed in the first half of 2021 (weeks 18–25/2021), followed by a decreasing trend in weeks 26–43/2021. For the weeks before and during the early rise of Omicron (44/2021–1/2022), we identified an increasing trend of 10.7% and for weeks 2–27/2022 we observed a decreasing trend of 18.1%. Unfortunately, we do not have available data about the vaccination status of the SARS-CoV-2 cases, ICU admissions or deaths. Our findings suggest that the Omicron variant is associated with increased transmissibility and reduced morbidity and mortality despite the previous increase in the trend of SARS-CoV-2 vaccinations.

## 1. Introduction

The Omicron SARS-CoV-2 variant (B.1.1.529) emerged in November 2021 as a variant of concern [1]. The Omicron variant contains 37 mutations in the spike protein and is characterized by an increased transmissibility that leads to high rates of SARS-CoV-2 spread [2]. On 2 December 20221, Greece recorded the first case of the Omicron variant, detected in a Greek citizen that had recently travelled to South Africa [3]. Due to the high spread of Omicron, the Delta variant was sharply displaced.

Since the announcement of the Omicron variant discovery and its fast identification in several countries around the world, many issues have arisen [4]. The major concern is the severity of the disease associated with the Omicron variant. Studies have suggested that vaccination provided protection from mortality and COVID-19 incidence during the eras of the Alpha and Delta variants [5]; however, there are a large number of infections with the B.1.1.529 variant occurring in people previously vaccinated against SARS-CoV-2 or that have a history of a SARS-CoV-2 infection [4]. Shortly after the emergence of the Omicron variant, there were reports suggesting that the B.1.1.529 variant is associated with a lower risk of severe COVID-19-related outcomes (such as hospitalizations) and reduced mortality in hospitalized patients [6,7]. In a recent report by the Centers for Disease Control and Prevention (CDC), the risk of severe COVID-19 in the USA was decreased in subjects infected by the Omicron variant [7]. Similarly, reports from England and South Africa report a lower risk of hospital admission in Omicron-infected individuals when compared to Delta variant infections [8,9].

The objective of the present study is to address differences in the rates of SARS-CoV-2 cases and COVID-19-related outcomes, in relation to SARS-CoV-2 vaccinations, between the periods that the different variants of SARS-CoV-2 prevailed (with special interest in the Omicron period), in Greece. To this end, we used national surveillance data in order to report the rate of SARS-CoV-2 cases, COVID-19-related ICU admissions and deaths, as well as the vaccinations against SARS-CoV-2 in Greece, during the time periods where the Alpha variant (B.1.1.7) vs. the Delta variant (B.1.617.2) vs. the Omicron variant (B.1.1.529) was prevalent.

## 2. Materials and Methods

### 2.1. Study Population

In our study, we used national surveillance data for SARS-CoV-2 cases, COVID-19-related ICU admissions and COVID-19-related deaths from 1 January 2021 to 10 July 2022. SARS-CoV-2 cases were defined as laboratory-confirmed SARS-CoV-2 cases (symptomatic or asymptomatic), COVID-19-related ICU admissions referred to patients admitted in the ICU with COVID-19 and deaths attributed to COVID-19 were defined as deaths in patients with confirmed COVID-19. The data were retrieved from the National Public Health Organization (NPHO) of Greece [10]. We analyzed the data according to the weekly prevalence of the main SARS-CoV-2 variants in Greece. The variants were identified with nucleic acid amplification tests (NAATs) based on reverse transcriptase PCR (RT-PCR). In more detail, we grouped data from the 2nd week of 2021 (starting at 7 January 2021) till the 26th week of 2021 (ending at 4 July 2021) where the Alpha variant (B.1.1.7) was prevalent (i.e., present in more than 50% of samples) (Period 1). The Delta variant was prevalent from week 27 of 2021 (5 July) till the 49th week of 2021 (19 December 2021) (Period 2) while the Omicron variant (B.1.1.529) was prevalent from the 50th week of 2021 (20 December 2021) till the end of the examined period (week 27 of 2022) (Period 3) [11]. Since age may influence the severity of COVID-19, we further stratified the population according to their age (<70 years and ≥70 years) [12]. In order to address the association of COVID-19 cases and outcomes with vaccination status, we use data concerning vaccinations from the European Centre for Disease Prevention and Control (ECDC) [13]. The data derived concerned fully completed vaccinations. The national vaccination program started with the Pfizer/BioNTech vaccine; the Oxford/Astra Zeneca, Moderna and Johnson & Johnson vaccines were subsequentially introduced [14].

Ethics approval was not applicable since we revised publicly available national surveillance data. We did not use personal identifications on demographic or personal data in the present study.

### 2.2. Statistical Analysis

Data are presented as absolute numbers or as percentages. We used a joinpoint regression model in order assess the variation in the trends of the rates of SARS-CoV-2 cases, COVID-19-related ICU admissions and COVID-19-related deaths, as previously described [14]. Briefly, this model investigates the combinations of trends that result in a statistically significantly better fit to a data series than a single-trend line fitted by Poisson regression or time-series models [15]. The Joinpoint Regression Program (3.5.2) and SPSS 20 were used to analyze the data. A statistically significant joinpoint was set at *p* < 0.05.

## 3. Results

**Figure 1:**
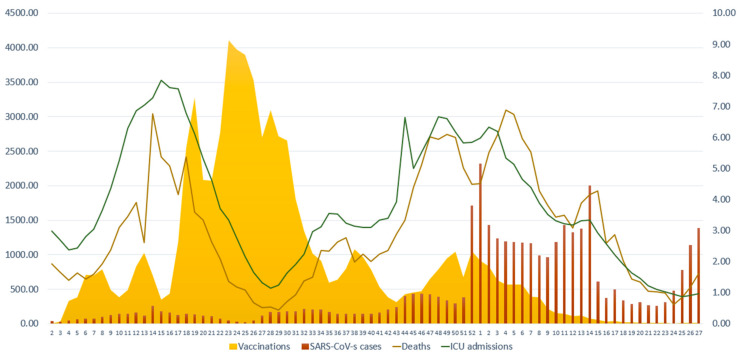
Number of new weekly SARS-CoV-2 cases, COVID-19-related ICU admissions, COVID-19-related deaths and vaccinations against SARS-CoV-2 during the study period. The scale for SARS-CoV-2 cases and vaccinations is on the left y axis (0–4500) and the scale for COVID-19-related ICU admissions and COVID-19-related deaths is on the right y axis (0–9). Data are expressed per 100,000 population. X axis corresponds to the number of weeks.

During the study period, we recorded 3,904,145 new SARS-CoV-2 cases, 25,213 deaths due to COVID-19 and 7,709,336 vaccinations against SARS-CoV-2 (Table 1 and Figure 1). During the weeks that the Alpha variant prevailed (Period 1), we observed 280,962 SARS-CoV-2 cases, 7474 COVID-19-related deaths and 3,978,634 vaccinations. During the period that the Delta variant prevailed (Period 2), there were 608,500 cases, 7154 deaths and 2,910,475 vaccinations. During the Omicron variant period (Period 3), we recorded 3,014,683 cases, 10,585 deaths and 820,227 vaccinations. We divided the absolute number of SARS-CoV-2 cases and COVID-19-related deaths to the number of weeks of each time period in order to roughly estimate the average weekly cases and deaths (Table 1). We observed that the average weekly cases were higher during the Omicron period (103,954.59 cases per week) followed by the period that the Delta variant prevailed (25,354.17 cases per week) and the Alpha variant period (11,238.48 cases per week). Average weekly vaccinations were higher during the Alpha period followed by the Delta period (Table 1). In the Omicron period, we recorded fewer weekly vaccinations (28,283.69 per week). The average weekly deaths were higher during Period 3 (365 deaths per week) followed by Period 1 (298.96 deaths per week) and Period 2 (298.08 deaths per week). Interestingly, weekly deaths in subjects <70 years were lower during the Omicron period (49.52 deaths per week) than during the Delta variant period (54.83 deaths per week) and the Alpha period (51.20 deaths per week). On the contrary, deaths in subjects ≥70 years were higher during the Omicron period vs. the Delta and Alpha periods (315.48 vs. 243.25 vs. 247.76 deaths per week, respectively).

We used joinpoint regression analysis to assess the variation in the trends of the rate of vaccinations, SARS-CoV-2 cases, COVID-19-related ICU admissions and COVID-19-related deaths. With this technique, we can identify the week (joinpoint) where a statistically significant abrupt change in temporal trends has occurred [15]. For vaccinations against SARS-CoV-2, the analysis identified 5 joinpoints leading to 6 periods where different trends were observed (Table 2). An increasing trend was observed in weeks 18–25 of 2021 (i.e., when the Alpha variant prevailed) with a 8.3% change (CIs: 2.0–15.0), which was followed by a decreasing trend (−11.9% change, CIs: −13.7–−10.1) during the weeks 26–43 of 2021. For week 44/2021 to week 1/2022, we identified a sharp increase in the trend of vaccinations by 10.7% (CIs: 2.7–19.3). The last period of the study (i.e., Omicron variant period) presented a 18.1% sharp decrease in the vaccination trend (CIs: −21.9–−14.0). For SARS-CoV-2 cases, the analysis identified 5 joinpoints that lead to 6 periods of different trends. The fastest increasing trend was observed in the weeks 50/2021 to 1/2022, which represent the rise in the Omicron wave (88.5% change, CIs: 6.5–233.6). There was a small increasing trend in weeks 4–14 of 2022 (4.2% change, CIs: 0.4–8.0), which was followed by a descending trend in the weeks 15–19 of 2022 (34.8% change, CIs: −47.1–−19.4). In the last period (weeks 20–27 of 2022), there was an increasing trend in cases by 28.3% (CIs: 20.1–37.2). For ICU admissions, we identified 5 joinpoints that correspond to 6 periods with different trends. For the first period (weeks 2–16 of 2021), we observed an increasing trend (10.4% change, CIs: 8.7–32.2) that was followed by a decreasing trend till week 29 of 2021 (−14.2% change, CIs: −16.1–−12.1). After a period of subtle stability in the trends (weeks 30–33), we identified an increasing trend during the weeks 34–50/2021 (5.7% change, CIs: 4.4–7.0). For the two following periods (week 51 of 2021 till week 16 of 2022 and weeks 17–27/2022), which correspond to the Omicron period, we observed a decreasing trend in the rate of ICU admissions, by −5.0% (CIs: −6.1–−3.9) and by −15.2% (−19.1–−11.2), respectively. As far as COVID-19-related deaths are concerned, the analysis identified 4 joinpoints with abrupt changes in the trends that lead to 5 periods with different trends. There was a fast increasing trend between weeks 2–17 of 2021 (10.8% change, CIs: 7.6–14.1) followed by a fast decreasing trend until week 29/2021 (−20.1%, CIs: −25.6−15.1). There was a slight increasing trend in deaths between the 34th week of 2021 and the 2nd week of 2022 (5.5%, CIs: 3.8–7.1). In the latest period of the study (week 5–27/2022), we observed a decreasing trend in deaths of 8.1% (CIs: −9.7–−6.6).

**Figure 2:**
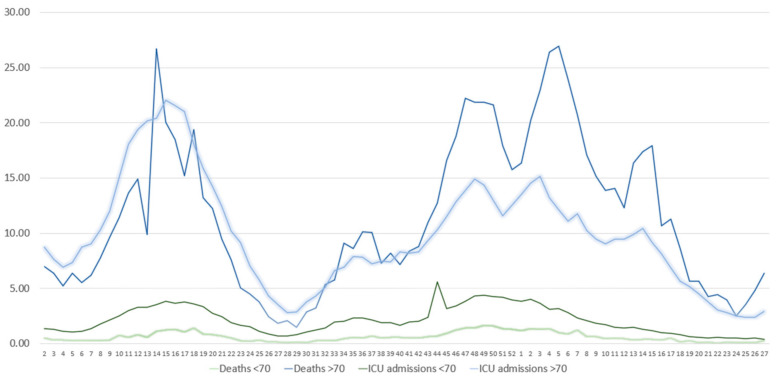
Absolute number of weekly COVID-19-related ICU admissions and COVID-19-related deaths in subjects <70 and ≥70 years. Data are expressed per 100,000 population. X axis presents the number of weeks.

Since COVID-19 outcomes are worse in the elderly [12], we stratified the population according to age (<70 years and ≥70 years) and performed joinpoint regression analysis to assess for differences in the trend of the rates of COVID-19-related ICU admissions and deaths (Figure 2). For SARS-CoV-2 cases in subjects <70 years, we observed a slight increasing trend during the Alpha and Delta waves (weeks 2–50/2021) (3.3% change, CIs: 2.5–4.1) (Figure 2). There was a major significant change during the rise of the Omicron wave (week 50/2021 to week 1/2022) by a 90.6% (CIs: 11.8–225.1) increase in the trend for cases, which was followed by a stability in the trend for the weeks 1–4/2022. After a slight increase by 3.6% (CIs: 0.0–7.4) during the weeks 5–14/2022, we observed a decreasing trend (−35.5% change, CIs: −47.9–−20.2) during the weeks 15–19/2022. During the last period of the study (weeks 20–27/2022), we identified a major increase in cases of 29.0% (CIs: 20.9–37.6). Cases in subjects ≥70 years followed a different pattern. For weeks 2–14 of 2021, we observed an increasing trend (15.9% change, CIs: 5.3–27.7) that was followed by a decreasing trend (−18.5%, CIs: −30.9– −3.8). We identified an increasing trend during week 23/2021 till week 13/2022 (8.7% change, CIs: 7.6–9.9), which was followed by a decreasing trend in weeks 14–22/2022 (−19.3% change, CIs: −25.0–−13.3). During the last weeks of the study (weeks 23–27/2022), we observed a major increasing trend in cases (51.4% change, CIs: 32.8–72.6), similar to what was identified in subjects ≥70 years. As far as COVID-19-related ICU admissions were concerned, we observed some similarities in the trends observed. In more detail, in subjects aged <70 years we identified an increasing trend of 10.1% (CIs: 7.5–12.9) during weeks 2–17/2021, a decreasing trend of −16.4% (CIs: −21.5–−11.1) for weeks 17–26/2021, an increasing trend for weeks 26–50/2021 (Delta wave) of 7.4% (CIs: 6.0–8.9) and finally a decreasing trend (−8.6%, CIs: −9.7–−7.5) for week 51/2021 till week 27/2022 (Omicron wave). For subjects aged ≥70 years, we identifed an increasing trend (9.9% change, CIs: 8.4–11.3%) during weeks 2–16/2021, a decreasing trend (−14.5%, CIs: −16.4–−12.5) for weeks 16–28/2021 and an increasing trend during weeks 28–48/2021 (6.9% change, CIs: 5.7–8.1). During the last two periods (week 49/2021 till 14/2022 and weeks 15–27/2022), we observed a decreasing trend in ICU admissions (−2.3%, CIs: −3.3–−1.3, and −11.3%, CIs: −13.5–−9.0, respectively). When assessing for differences in the trends of COVID-19-related deaths between patients <70 and ≥70 years, we observed disparities during the Alpha and Delta waves, while during the Omicron wave we identified similarities, at least in the direction of the trend. To streamline our observations, deaths of subjects <70 years displayed a decreasing trend during weeks 2–14/2021 (−10.7% change, CIs: −18.7–−1.9), which was followed by an increasing trend during weeks 15–23/2021 (48.0% change, CIs: 36.2–60.7). After a period of subtle stabiblity in the trend until week 33, we identified a decreasing trend till the end of the study. We observed a −2.8% change (CIs: −2.8–−4.2) during week 34/2021 till week 2/2022 and a −20.1% change from week 3/2022 till week 27/2022 (CIs: −25.1–−14.7). For deaths in subjects ≥70 years, joinpoint analysis did not identify significant differences in the trends from week 2/2021 till week 19/2021 (Alpha period). From weeks 20–42/2021, we observed a decreasing trend (−17.2%, CIs: −19.5–−14.8), followed by an increasing trend till week 01/2022 (28.5% change, Cis: 13.4–45.6). For the weeks 2–27/2022, we identified a decreasing trend of −20.9% (CIs: −26.5–−14.7).

## 4. Discussion

In the present ecological study, we observed that during the first weeks that the Omicron variant prevailed in Greece there was a sharp increase in SARS-CoV-2 cases with an 88.5% change in the trend. The increasing trend in the rate of cases remained in the majority of weeks of the Omicron period despite the fact that the vaccinations trend in the previous period was increasing. The ICU admissions trend decreased immediately after the emergence of Omicron in Greece while deaths displayed a decreasing trend a few weeks later. Our data strengthen the concept that the Omicron variant displays increased transmissibility and is associated with a decreased severity of COVID-19 when compared to the Delta and Alpha variants. To our knowledge, this is the first study reporting data of Omicron-related severity at the national level of Greece.

Our data support the notion that the Omicron variant is more transmissible than the Delta or the Alpha variants. We observed a sharp and abrupt increase in SARS-CoV-2 cases of 88.5% during the rise of the Omicron wave despite the increased trend in vaccinations in the immediate period before the Omicron outbreak. A similar pattern of exponential increase in new SARS-CoV-2 cases during the Omicron variant outbreak has been reported in South Africa [16]. The Omicron variant has several mutations, which increase its infectivity since they may result in an increased tightness and affinity to the angiotensin-converting enzyme 2 (ACE2) receptor and/or more rapid cell-to-cell fusion [17]; mutations in the receptor binding protein of the S-protein (such as G339D, S371L, S373P, S375F, K417N, N440K, G446S, S477N, T478K, E484A, Q493R, G496S, Q498R, N501Y, Y505H) have been associated with greater affinity to the ACE2 receptor and may lead to the enhanced Omicron transmissibility [18]. A 13-fold increase in viral infectivity has been reported compared to the Delta and Alpha variants [19]. An explanation may be the variant’s ability to infect vaccinated or previously exposed subjects [20]. Additionally, one has to take into account that the protection of the two shots of vaccines falls readily after a few months while the third dose may increase the protection against severe COVID-19 [21,22]. The increase in cases despite the previous increase in vaccinations is underlined by the reported virus escape from vaccine-induced antibodies. Omicron is associated with 41–84% less neutralization of antibodies when compared to the Delta variant [23]. In addition, the substitution S:655Y, which is present in the Omicron variant, results in enhanced viral replication and spike protein cleavage and may partially underline the variant’s increased transmissibility [23]. Furthermore, Omicron has a shorter doubling time (between 2–3 days), which may contribute to the high rise of the cases [24]. The high rise in cases may be partially explained by social conditions since the time point of the rise of Omicron corresponds to the Christmas season.

During the rise of the Delta wave, we identified an increase in ICU admissions and deaths. On the contrary, ICU admissions displayed a decreasing trend almost immediately after the rise of Omicron in Greece. Similarly, deaths had a decreasing trend a few weeks later, which was evident in both age groups (<70 and ≥70 years). A reduced rate of hospitalization during the Omicron wave has been reported in South Africa [16]. Ulloa et al. reported a 65% reduced risk of hospital admission or death in patients affected by Omicron vs. the Delta variant in Canada [2]. Similarly, in the United Kingdom, the risk of death was significantly reduced by 65% for Omicron vs. the Delta variant [25]. In the same context, the mortality rate of COVID-19 decreased for all patient groups during the Omicron period vs. Delta in the USA [6]. Similar results have been reported by others [26]. One of the potential reasons for the fall in the severity of COVID-19 during Omicron may be the fact that Omicron replicates mainly in the upper airways thus leaving the lower airways unaffected. The reason(s) underlying the reduced incidence of lower tract involvement with Omicron are not well understood. Studies have shown that Omicron replicates less in lung cells vs. bronchial cells [27,28]. The mutations of the Omicron variant may result in affected receptor engagement, cell and tissue tropism, or altered temperature sensitivity, which may lead to the reduced pathogenicity of lower tract [28].

The magnitude of the severity of COVID-19 following infection with Omicron has been reported to be age-dependent. Nyberg et al. reported that severity reduction is lower for subjects older than 80 years than younger subjects [26]. In our study, as far as ICU admissions are concerned, we observed that in both age groups there was a decreasing trend after the Omicron outbreak. For <70 years, there was a −8.6% trend. For ≥70 years, the trend had two joinpoints; for the first 15 weeks of Omicron there was a −2.3% trend that was followed by the sharpest decrease in the trend of −11.3%. However, for deaths there was a similar decreasing trend in both groups that occurred later (week 6 vs. week 2) on subjects <70 years vs. ≥70 years, respectively. In the rise of Omicron, some disparities occurred between the deaths of subjects <70 and ≥70 years, which may be attributed to some deaths occurring from the Delta variant.

Our study is not without limitations. A major limitation of the study is that we do not report data about the vaccination status/type of vaccine administered of the SARS-CoV-2 cases, ICU admissions or deaths, or the individual viral strain of the cases since those data were not available to us. In the same context, we do not have available data about other possible cofounders, such as gender or comorbidities, that could affect the severity of COVID-19. Another possible limitation is the fact that the analysis was performed in national surveillance data. Although the examination of publicly available data is commonly used in research since it could help answer research questions that concern national responses to SARS-CoV-2, there are some limitations that are attributed to the ecological methodology. The design of the present study cannot assess the potential impact of non-pharmaceutical interventions or individual factors that could influence the exposure–response association. We must also acknowledge as a limitation of the present study the fact that it occurred before the authorization of the adapted vaccines; therefore, our results cannot be extrapolated for the Omicron-specific vaccines.

## 5. Conclusions

In conclusion, our findings support the concept that the Omicron variant, when compared to the Delta or Alpha variants, is associated with increased transmissibility and reduced morbidity and mortality. Now that the pandemic is close to becoming an endemic, it may be important to continue monitoring the infection rates, the effectiveness of the adapted vaccines and the possible emergence of genetic variants (through various surveillance systems), in order to enable the development of effective public health interventions [29]. Future studies should concentrate on the deployment of COVID-19 therapy aiming at reducing virus load and therefore transmissibility, and measures should be taken to embrace vaccination campaigns for adapted vaccines [30].

## Figures and Tables

**Table 1:** Total and weekly average of SARS-CoV-2 cases, COVID-19-related ICU admissions, COVID-19-related deaths and vaccinations. Data are expressed as absolute numbers.

Period	New SARS-CoV-2 Cases			Average New SARS-CoV-2 Cases per Week			ICU Admissions			Average ICU Admissions per Week			Deaths			Average Deaths per Week			Vaccinations			Average Vaccinations per Week		
	All Ages	<70 Years	≥70 Years	All Ages	<70 Years	≥70 Years	All Ages	<70 Years	≥70 Years	All Ages	<70 Years	≥70 Years	All Ages	<70 Years	≥70 Years	All Ages	<70 Years	≥70 Years	All Ages	<70 Years	≥70 Years	All Ages	<70 Years	≥70 Years
Period 1 (2nd w. of 2021 to 26th w. 2021)	280,962	240,082	40,880	11,238.48	9603.28	1635.20	12,494	4805	7689	499.76	192.20	307.56	7474	1280	6194	298.6	5120	24,776	3,978,634	1,821,690	2,156,944	159,145.36	72,867.60	86,277.76
Period 2 (27th w. of 2021 to 50th w. of 2021)	608,500	548,398	60,102	25,354.17	22,849.92	2504.25	9495	4801	4694	395.63	200.04	195.58	7154	1316	5838	29.808	5483	24,325	2,910,475	2,552,534	357,941	121,269.79	106,355.58	14,914.21
Period 3 (51st w. of 2021 to 27th w. of 2022)	3,014,683	2,681,109	333,574	103,954.59	92,452.03	11,502.55	9997	4260	5737	344.72	146.90	197.83	10,585	1436	9149	365.00	49.52	315.48	820,227	469,674	350,553	28,283.69	16,195.66	12,088.03
Total	3,904,145	3,469,589	434,556	140,547.23	124,905.23	15,642.00	31,986	13,866	18,120	1240.11	539.14	700.97	25,213	4032	21,181	962.04	155.55	806.49	7,709,336	4,843,898	2,865,438	308,698.84	195,418.84	113,280.00

Abbreviations: w, week; ICU, intensive care unit.

**Table 2:** Joinpoint regression analysis of trends in SARS-CoV-2 cases, ICU admissions and deaths stratified by age group. * *p* < 0.05.

	SARS-CoV-2 Cases		ICU Admissions			Deaths
	Period (Week)	%Change (CIs)	Period (Week)	%Change (CIs)	Period (Week)	%Change (CIs)
**All ages**	2–50/2021	3.1% * (2.3–4.0)	2–16/2021	10.4% * (8.7–12.2)	2–17/2021	10.8% * (7.6–14.1)
	51/2021–1/2022	88.5% * (6.5–233.6)	17–29/2021	−14.2 * (−16.1–−12.1)	18–29/2021	−20.5% * (−25.6–0–15.1)
	2–4/2022	−24.8% (−49.2–0–11.2)	30–33/2021	21.3% (−5.1–55.1)	30–34/2021	41.1% (−6.2–112.3)
	5–14/2022	4.2% * (CIs: 0.4–8.0)	34–50/2021	5.7% * (4.4–7.0)	35/2021–2/2022	5.5% * (3.8–7.1)
	15–19/2022	−34.8% * (−47.1–−19.4)	51/2021–16/2022	−5.0% * (−6.1–−3.9)	3–27/2022	−8.1% * (−9.7–−6.6)
	20–27/2022	28.3% * (CIs: 20.1–37.2)	17–27/2022	−15.2% * (−19.1–−11.2)		
**<70 years**	2–50/2021	3.3% * (2.5–4.1)	2–17/2021	10.1% * (7.5–12.9)	2–14/2021	10.7% *(−18.7–−1.9)
	50/2021–01/2022	90.6% * (11.8–225.1)	18–26/2021	−16.4% * (−21.5–−11.1)	15–23/2021	48.0% * (36.2–60.7)
	2–4/2022	−25.6% (−48.7–7.7)	27–50/2021	7.4% * (6.0–8.9)	24–30/2021	−0.5% (−5.5–4.8)
	5–14/2022	3.6% * (0.0–7.4)	51/2021–27/2022	−8.6% * (−9.7–−7.5)	31–33/2021	−31.5% (−54.6–3.4)
	15–19/2022	35.5% * (−47.9–−20.2)			34/2021–5/2022	−2.8% * (−2.8–−4.2)
	20–27/2022	29.0% (20.9–37.6)			6–27/2022	−20.1% * (−25.1–−14.7)
**≥70 years**	2–14/2021	15.9% * (5.3–27.7)	2–16/2021	9.6% * (8.4–11.3)	2–6/2021	295.5% (−43.2–2654.8)
	15–23/2021	−18.5% * (−30.9–−3.8)	17–28/2021	−14.5% * (−16.4–−12.5)	7–16/2021	1.3% (−6.0–9.1)
	24/2021–13/2022	8.7% * (7.6–9.9)	29–48/2021	6.9% * (5.7–81.1)	17–19/2021	62.6% (−3.1–172.8)
	14–22/2022	−19.3% * (−25.0–13.3)	49/2021–14/2022	−2.3% * (−3.3–−1.3)	20–42/2021	−17.2% * (−19.5–−14.8)
	23–27/2022	51.4% * (32.8–72.6)	15–27/2022	−11.3% * (−13.5–−9.0)	43/2021–1/2022	28.5% * (13.4–45.6)
					2–27/2022	−20.9% * (−26.5–−14.7)

## Data Availability

Data are available upon request.
